# Comparison of NF-RO and RO-NF for the Treatment of Mature Landfill Leachates: A Guide for Landfill Operators †

**DOI:** 10.3390/membranes8020017

**Published:** 2018-03-21

**Authors:** Sreenivasan Ramaswami, Joachim Behrendt, Ralf Otterpohl

**Affiliations:** Institute of Wastewater Management and Water Protection, Hamburg University of Technology (TUHH), Eissendorfer Str. 42, 21073 Hamburg, Germany; j.behrendt@tuhh.de (J.B.); ro@tuhh.de (R.O.)

**Keywords:** landfill leachate, monovalent ions, nanofiltration, organics, reverse osmosis

## Abstract

Reverse osmosis (RO) and nanofiltration (NF) are among the state-of-the-art technologies for treating landfill leachates. Due to the complexity and variance in the composition of leachates, numerous combinations of multiple technologies are used for their treatment. One process chain for the treatment of raw leachate is RO followed by further concentration of RO-retentate using NF (RO-NF scheme). The aptness of this process train used by some landfill sites around the world (usually with the aim of volume reduction so as to re-inject the concentrate into the landfill) is questionable. This study investigated two schemes RO-NF and NF-RO (nanofiltration of raw leachate followed by reverse osmosis of NF permeate) to identify their merits/demerits. Experiments were conducted in bench scale using commercial membranes: DOW Filmtec NF270 and SW30HR. Filtration trials were performed at different pressures to compare the water and solute transports in the individual stages of the two schemes. Based on the water fluxes and compositions of retentates and permeates; osmotic pressures, energy demands, and other possible operational advantages were discussed. NF-RO offers some advantages and flexibility for leachate treatment besides being energy efficient compared to RO-NF, wherein osmotic pressure steadily increases during operation in turn increasing operation and maintenance costs.

## 1. Introduction

Landfill leachates are one among the most polluted wastewaters, characterized by high chemical oxygen demand (COD) and ammonia content. The characteristics of organics and their concentration, as well as the concentration of N-NH_4_^+^ depends on the landfill age [1]. Additionally, depending on the type of waste deposited, they can contain high concentrations of heavy metals (e.g., Zn^2+^, Cr^3+^, Ni^2+^, Cd^2+^, Pb^2+^, As^3+^, or their speciations), other metal ions (Na^+^, K^+^, Ca^2+^, Mg^2+^, Fe^2+^, and Al^3+^) and anions like Cl^−^, SO_4_^2−^, PO_4_^3−^, etc. Numerous technologies, in many possible combinations of them, have been studied and used for leachate treatment [2,3,4].

Reverse osmosis is a state-of-the-art technology for the treatment of landfill leachates. RO process (using cellulose acetate membrane) was studied for treatment of landfill leachate within two decades from its discovery and was reported to be the most effective method for treating leachates [5]. By the mid-1980s, RO systems had already penetrated significantly into the market of leachate treatment [6,7]. The technology is highly promising and efficient for the treatment of landfill leachates having rejection coefficients on an average greater than 0.98 [3,6,8,9] for all pollutants (esp. organics and heavy metals), thus providing high quality permeate. RO systems were installed in more than 100 landfill sites in northern Europe, North America, and the Far East during the last decade [10]. 

Recirculation of leachate constituents by controlled re-injection of RO concentrate into the landfill has been researched and practiced widely. It has been shown to enhance the biodegradation rate or landfill gas production in young landfills, which contain a high portion of biodegradable organic matter [11,12,13,14]. However, it is also reported that constituents like ammonia, chloride, and metals are not removed by recirculation [11,15], due to which this cannot be a sustainable solution. Therefore, re-injection of RO retentate cannot be beneficial in the case of old landfills producing methanogenic leachate (containing no biodegradable COD) and would likely result in an increase in strength of the leachate. For this reason, recirculation of methanogenic leachates is forbidden by the German Landfill Ordinance 2009, unless the landfill operator can prove the gain of any benefit from doing so [16].

**Figure 1:**
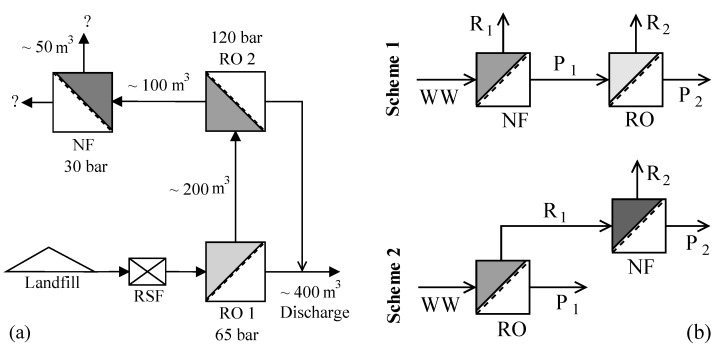
(**a**) Treatment scheme at landfill site (based on IAG [17] and Rautenbach et al. [19]) (RSF—rapid sand filtration); (**b**) Integration options investigated in this study (WW—wastewater/leachate, R—Retentate, P—Permeate).

The leachate considered in this study is from the hazardous waste landfill site at Ihlenberg, Germany; one of the largest and most advanced landfills in Europe [7]. The site with a landfill area of 113 hectares (1.13 km^2^) was commissioned in 1983 with a total capacity of 26 million cubic meters [17]. By the end of 1989, an RO system with 36 m^3^/h capacity was installed for treating the raw leachate, recovering up to 80–85% clean water and re-injecting the retentate [7]. Some studies at the Ihlenberg dumpsite in the past [7,9,18,19] have focused on minimizing the concentrate volume to be re-injected, as retentate disposal is very cost intensive. Some of them investigated the application of reverse osmosis at 200 bar while some investigated a combination of RO, high pressure RO and high pressure NF (as in Figure 1a) for reducing the volume of concentrate as low as possible. To comply with the ban on retentate recirculation, the RO concentrate currently gets treated further as in Figure 1a, the aptness of which (the process train) is questionable. 

This study investigates the two integration options: NF-RO and RO-NF for this scenario, as illustrated in Figure 1b. Scientists involved in the field of membrane technologies would typically recommend NF-RO over RO-NF [20,21]. However, landfill operators and leachate professionals, who do not necessarily possess a good knowhow of nanofiltration and reverse osmosis processes, will not be in a position to analyze these two schemes. The practice of nanofiltration of RO concentrate has been reported in literature [19,22] for landfill sites like Halle Lochau (Germany), Goerlitz (Germany), and Yachio Town (Japan). The first author of this paper came to know of similar other cases at the Sardinia Symposium 2017 (personal communication, leachate professional).

NF compared to RO can be operated at lower pressures; offers higher fluxes; rejects organics and multivalent ions selectively; and incurs lower investment, operation, and maintenance costs. Due to these reasons, numerous studies in the recent past have preferred NF over RO and explored its ability for the treatment of landfill leachates [6,23,24,25,26,27,28,29,30,31]. However, no study could be identified, which investigated a combination of NF and RO (NF-RO) for treating landfill leachates. Integration of technologies has been emphasized for achieving efficient and economic treatment of landfill leachates [24,28,32,33,34,35], which could also offer more flexibility and advantages for the individual processes in the process chain [35].

This study aims to investigate the two combinations (NF-RO and RO-NF) for the treatment of old landfill leachates, and to identify the merits/demerits of the two process trains. Experiments were conducted in bench-scale to demonstrate the differences between the two schemes, with respect to water fluxes (energy requirements), rejection of solutes, and other operational advantages. This paper critically analyses the two integration options, and discusses some fundamental properties and transport characteristics of nanofiltration and reverse osmosis membranes applied to leachate treatment.

## 2. Materials and Methods

The experiments were carried out in the laboratories for drinking water research at the Institute of Water Resources and Water Supply, TUHH. For this reason the wastewater was prepared synthetically by dissolving known weights of the compounds (as in Table 1) in deionized water to have a composition resembling that of the raw leachate from the Ihlenberg landfill site (Table 2). This was done to avoid any kind of contamination of the laboratories from the hazardous landfill leachate. Sodium salt of humic acid, purchased from Carl Roth GmbH (Karlsruhe, Germany), had a humic acid (HA) content of about 45–65%. The HA salt had a sodium content of about 8–9% as Na_2_O (Carl Roth GmbH, personal communication). The HA salt was measured to have a total organic carbon (TOC) content of 300 mg/g. All other solutes used were of analytical grade.

The HA salt had not dissolved completely, even over several hours of stirring. The synthetic leachate was filtered using a folded filter paper (Carl Roth 600P-500, 13 µm pore diameter) to remove the undissolved HA salt. The filtrate was analyzed for N-NH_4_^+^; total inorganic carbon (TIC), TOC, and COD concentrations; absorbance at 254 nm (Abs254); and conductivity, and used for all the experiments.

**Figure 2:**
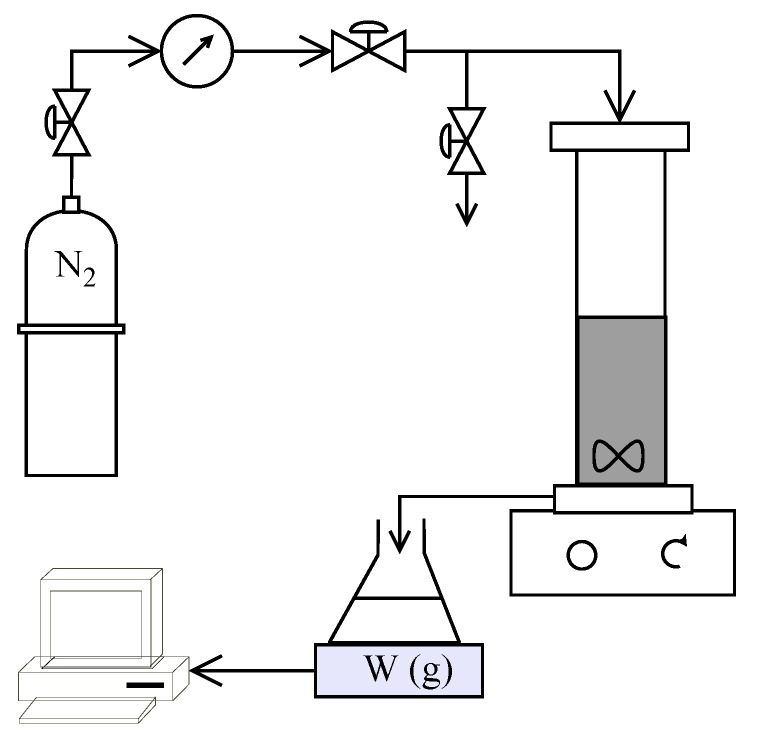
Schematic of experimental setup used in the study.

A schematic of the experimental setup used in the study is shown in Figure 2. HP4750 Stirred Cell (Sterlitech Corporation, Kent, Washington, DC, USA) was used to carry out the filtration trials in dead-end mode providing an active membrane area of 14.6 cm^2^. Nitrogen gas flowing through a digital manometer (Pressure Gauge Digital with Ceramic Sensor Element, Battery Powered MAN-SD from Kobold Messring GmbH, Hofheim, Germany) and pressure regulators (Swagelok, OH, USA) was used to apply the desired pressure inside the cell. The weight of the collected permeate was measured using an Acculab ATL-2202 balance (Sartorius AG, Goettingen, Germany) and recorded every 10 s. All experiments were carried out at ambient temperature (22 ± 1 °C) The recorded volume flow rate was used to estimate the temperature corrected permeate flux (using GE Water [36]) at 25 °C.

Polyamide thin film composite membranes Dow Filmtec SW30HR and NF270 were used for performing the reverse osmosis and nanofiltration experiments, respectively. The membranes were cut in circles of 50 mm diameter and stored in pure water (type 1, 0.05 µS/cm conductivity, produced using a Direct-Q^®^ 5UV-R system, Merck Millipore, Darmstadt, Germany) at 4 °C for more than 24 h to allow swelling. Two types of experiments were conducted, as illustrated in Figure 1b, to study the individual processes and their combination for treating the synthetic leachate. A total of eight trials were carried out as in Table 3 using virgin membranes for each experiment. Before each experiment, pure water was filtered through the respective membrane at the desired operating pressure + 5 bar (OP + 5 bar) until a steady permeate flux was achieved. This would result in a stable membrane structure (after subjecting the membrane to possible compaction at that pressure) before operation and also enable the determination of pure water permeability of the membranes.

The trials were planned such that about 50% water recovery was achieved from each stage of the schemes. Feed volume for second stage in each scheme was fixed at 100 mL. Therefore, the first stage was operated so as to obtain 120 mL of desired feed (NF permeate in scheme 1 or RO retentate in scheme 2) for the second stage. 20 mL of the collected 120 mL was reserved for making the analyses. Samples of the different streams (F, R, and P) from each experiment were analyzed for N-NH_4_^+^, TIC, and TOC concentrations; Abs254; and conductivity. All analyses were carried out as in Table 4 following the German standard methods [37]. The parameters chosen to be analyzed would enable the characterization of rejection capacities of the membranes for organic (using TOC and Abs254 values) and inorganic solutes (using conductivity and N-NH_4_^+^ values).

## 3. Results and Discussion

### 3.1. Comparison of Water Transport

Table 5 shows the measured values for temperature corrected pure water fluxes (J_w_) and permeabilities (K_w_) for NF270 and SW30HR membranes at steady operating conditions. The effect of operating pressure (OP) on membrane compaction and the resulting negative impact on water permeability can be seen in Table 5. Several other studies [19,38,39,40] have reported decrease in water permeabilities due to membrane compaction with increase in operating pressure. The clean water permeability of NF270 membrane observed in this work is consistent with the values found in literature [41,42].

Using the experimentally determined values for pure water permeability and initial permeate flux of raw leachate with SW30HR membrane at respective operating pressures, the osmotic pressure (π) of raw leachate was calculated to be 14.36 ± 0.24 bar (using the relation π = OP − J_w_/K_w_). A theoretical approximation for osmotic pressure contributions from: inorganic electrolytes (Table 1) using van’t Hoff’s formula (π = Σ nRTC_s_ where C_s_ is electrolyte concentration in mol/m^3^, n is the no. of ions contained in the electrolyte of interest, R = 8.3142 J·K^−1^·mol^−1^ and T = 298.15 K) [43], and that from organic solutes using the empirical relation (π = 0.00311 × COD × 1.01325) reported by Chianese et al. [8] and the measured COD of raw leachate = 980 ± 23 mg/L gives 9.83 bar and 3.09 bar, respectively, totaling to 12.92 bar. 

**Figure 3:**
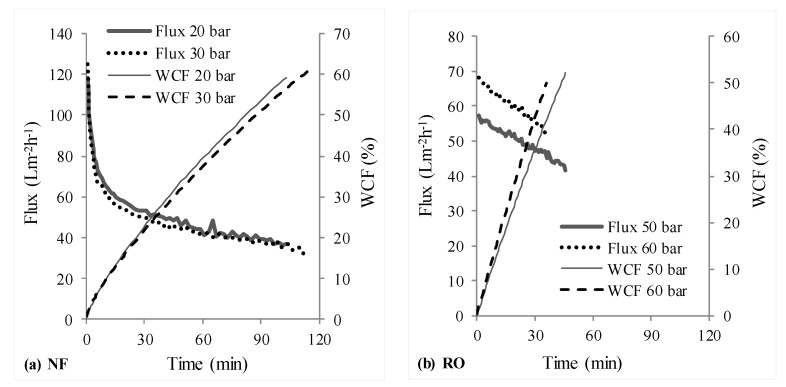
Measured permeate flux and WCF-NF followed by RO (scheme 1, NF → RO).

**Figure 4:**
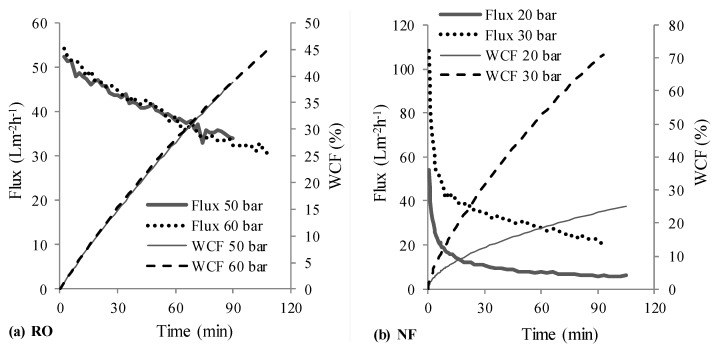
Measured permeate flux and WCF-RO followed by NF (scheme 2, RO → NF).

Figure 3 depicts the permeate fluxes and water conversion factors (WCFs) obtained at different operating pressures for NF and RO stages in scheme 1, wherein the NF stage treated the synthetic raw leachate and its NF permeate was treated by RO. Figure 4 shows the determined permeate fluxes and WCFs with scheme 2, wherein the synthetic raw leachate was first treated by RO and the RO retentate was further handled using NF. 

The initial sharp decrease in flux during nanofiltration (see Figure 3a and Figure 4b) compared to RO stages should be attributed to concentration polarization (and subsequent fouling) resulting from the high initial permeate fluxes offered by NF membrane; whereas the observed gradual decline in flux thereafter is due to increase in osmotic pressure of the solution in the filtration cell. Due to the initial high permeate fluxes and thus, high rejection rates of organics (i.e., entities (organic molecules/ions) rejected per unit time), combined with the fact that the trials were conducted in deposition mode—with limited ability of the stirrer inside the filtration cell to realize effective transport of rejected entities from the membrane surface to the bulk, it is likely that concentration polarization resulting in higher fouling tendencies caused the observed initial sharp decline. On the other hand, due to the fact that the permeate fluxes in the RO stage of RO-NF were lower in comparison, a sharp initial flux decline was not observed.

NF and RO stages in schemes 1 and 2, respectively, treated raw leachate which has a large amount of organics, whose contribution to osmotic pressure at similar concentrations is much larger than that of inorganic salts [8]. It is interesting to note that the difference between permeate fluxes achieved with higher and lower operating pressures in Figure 3a and Figure 4a is marginal, compared to the expected and marked difference to be seen in Figure 3b. Although in both these cases, the permeate fluxes were initially slightly higher at higher operating pressures (in comparison to lower operating pressure), they gradually became smaller. This is likely due to a combination of two reasons: membrane compaction (due to compaction, water permeability is lowered, the flux does not increase linearly with increase in operating pressure) and increased concentration polarization (and thus increased deposition or fouling) due to high TOC content in feed (organics concentration on the surface of the membrane operated with higher pressure can be higher, since the permeate flux was initially slightly higher for higher pressure).

On the other hand, a large difference in permeate fluxes at 20 and 30 bar pressures was to be seen in the NF stage treating RO concentrate (see Figure 4b). This should be due to the fact that the osmotic pressure of RO retentate would have been almost twice of that of the raw leachate. Osmotic pressure for nanofiltration will be lower than that for reverse osmosis, if some solutes in the feed can permeate the NF membrane [44]. As a result, the driving force (OP-π) would be smaller for operation at 20 bar than at 30 bar. Consequently, lower fluxes were recorded with 20 bar. Furthermore, it is interesting to see that the permeate flux (after the initial steady decline) at 30 bar in Figure 4b (from 40 to 20 L·m^−2^·h^−1^) is roughly 50–70% of that in Figure 3a (from 60 to 40 L·m^−2^·h^−1^). This supports the notion that the osmotic pressure of RO retentate in scheme 2 was roughly two times that of raw leachate.

Overall, permeate fluxes from each stage in scheme 1 were clearly higher than that obtained with scheme 2, which can be seen from Figure 3 and Figure 4. For NF and RO operated with 30 and 50 bar pressures respectively, a total of 176 mL permeate was recovered from both stages (Table 3) with scheme 1 in about 160 min, whereas it took about 200 min for recovering 162 mL permeate with scheme 2. This would translate to lower energy (or operating cost) and membrane area (or time) requirements as advantages of scheme 1 over scheme 2.

### 3.2. Comparison of Solute Transport

Table 6 and Table 7 show the measured values for the different parameters analyzed in the feed, retentate, and permeate samples from the experiments in schemes 1 and 2, respectively. TOC was measured by the difference method. Therefore, the measured TOC concentrations in permeate samples may not be very accurate (as TIC > TOC) [37]. Absorbance at 254 nm can be used as surrogate for TOC concentrations [45] in permeate samples. Abs254 values of samples expected to contain high TOC concentrations (raw leachate, all NF retentates, and RO retentates from scheme 2), were measured after diluting them 231 times so as to avoid sub-estimation of absorbance.

NF270 is a loose hydrophilic membrane with a molecular weight cut-off of about 200 Da and a high water permeability, showing high and low-to-high rejection capacities for organic solutes and inorganic ions, respectively [41,46,47,48]. These reasons explain the relationships to be seen between the values for different parameters measured in feed, retentate and permeate samples (Table 6 and Table 7) which correspond to the concentration factors (ratio of feed volume to retentate volume) achieved in the respective trials (see Table 3).

For instance, TOC and Abs254 values in NF permeate (at 30 bar in scheme 1) were quite low since NF270 can reject organics effectively and in NF retentate were roughly 2.5 times the values measured in raw leachate, comparable to the concentration factor of 2.6 obtained in that trial. On the other hand, for the same experiment, rejections for N-NH_4_^+^ and TIC (predominantly HCO_3_^−^) ranged from 20% to 60%, consistent with rejection capacities of NF270 for monovalent ions reported in literature [48,49,50]. Similarly, the measured values for different parameters in RO retentate and permeate also matched the concentration factors and expected rejections to a good extent.

It is to be emphasized that sharp demarcations and accurate estimations for rejection of solutes cannot be made from these experiments, since they were performed in dead-end mode, in bench scale, and as single trials. However, the aim of these measurements has not been to just characterize the rejection capacities accurately, but also to discuss the potential merits or demerits of the two integration options.

### 3.3. NF-RO vs. RO-NF

**Figure 5:**
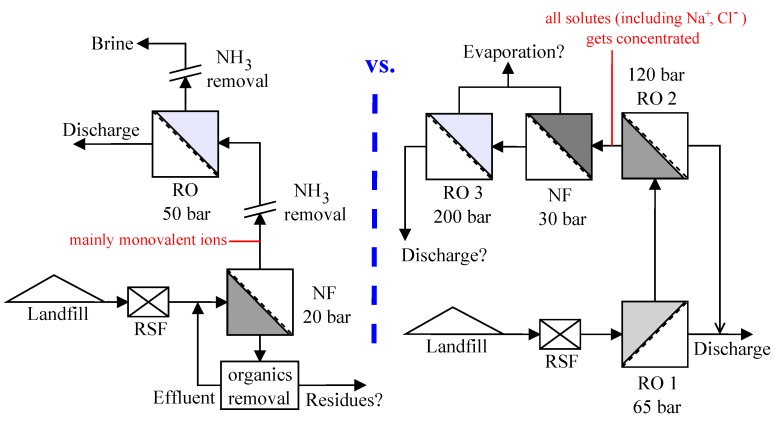
Comparison of: NF-RO—plausible operating pressures and perspectives on integration options (**left**); and process chain at the landfill site for leachate treatment using RO-NF (**right**).

Figure 5 compares some treatment perspectives for the NF-RO scheme with the prospective upgrade (based on [18]) of the leachate treatment system using RO-NF at the Ihlenberg landfill site. The schematic illustrates the potential options for combining different treatment methods and distinguishes the advantages of NF-RO compared to RO-NF strategy—as per the aim of this work. It can be seen (also from Table 6) that NF in scheme 1 facilitates the fractionation of pollutants into two streams—permeate stream containing mostly monovalent ions and retentate stream enriched with organic pollutants. As also reported by some other studies [6,51,52], relatively harmless monovalent ions like Na^+^ and Cl^−^, which are present in significant amounts in landfill leachates, can permeate an NF membrane; while toxic solutes like heavy metals and organic compounds are mostly retained. Due to these reasons, osmotic pressure of each stage in NF-RO is lesser compared to that in RO-NF, which explains the observed superior permeate fluxes (Figure 3 and Figure 4) in the former. 

On the other hand, in RO-NF the raw leachate is just concentrated several times leading to an increase in osmotic pressure necessitating the use of higher operating pressures, making it energy intensive. Integration of NF with RO (NF-RO) has been reported to be “much more energy-efficient” and to significantly reduce the costs for desalination processes compared to RO alone [20,21]. There is a pressing need for an energy-efficient system as leachate treatment is a long-term process. A closed landfill generates leachate for a timeframe of minimum 30 years, which can be as high as a couple of centuries [1,53,54]. The cost for leachate treatment in Germany has been evaluated to be in the range €10–70 per m^3^ leachate [55], which forms about 25–30% of the total aftercare costs [53].

High-pressure membrane processes alone cannot treat landfill leachates completely and efficiently [23,28,31,32,56,57], since NF and RO only enrich most of the contaminants in the retentate stream. In other words, a combination with other physical-chemical and/or biological processes is necessary for the removal or degradation of the contaminants. From this point of view, NF placed before RO can offer some other advantages, besides the reduction in energy requirements, operation and maintenance costs. 

Nanofiltration placed before reverse osmosis can provide greater flexibility for ammonia removal, since the organic compounds can be selectively separated. Ramaswami et al. [35] reported the ability to recover ammonia from nanofiltration permeate of raw leachate as clean and useable struvite, which can be sold as a fertilizer. Since organics and hardness contents in the NF permeate are low, stripping of ammonia from RO retentate of NF permeate can be accomplished without significant foaming and scaling problems, which is often faced during ammonia stripping from leachates [58,59,60]. Should biological ammonia removal from raw leachate be challenging due to inhibition from organics [61,62,63], nanofiltration might provide a solution to this scenario offering a less complex permeate for biological treatment. Organic pollutants which are enriched in the NF retentate can be treated appropriately using other physical-chemical processes such as coagulation/electrocoagulation-flocculation, ozonation followed by biological treatment, etc. [3,31,33,56,64]. 

On the other hand with RO-NF scheme, despite the requirement for higher operating pressures, the possibility for integration with other physical-chemical or biological processes becomes challenging or limited. At the Ihlenberg landfill, the raw leachate (see composition in Table 2) gets about five times concentrated by the two-stage reverse osmosis (about 30, 15, 4, and 3 g/L Cl^−^, Na^+^, TOC and NH_4_^+^-N, respectively, in RO retentate). For this scenario for instance, biological treatment of RO retentate or its NF permeate would be challenging due to high salinity and the purity of struvite precipitated from the NF permeate of RO-NF would be lower [35].

Investigation of fouling/scaling tendencies was not in the scope of this study and needs to be addressed in larger scale (in lab or pilot-scale) under real-time working conditions (with the actual landfill leachate in crossflow mode). It may be however, said in general that the fouling and scaling of RO membrane in NF-RO would be reduced (since the NF stage can remove most of the humic substances, and Ca^2+^, SO_4_^2−^, and other di/multivalent ions) compared to that in RO-NF. It is recommendable to apply viable pre-treatment or integration strategies for also minimizing the fouling of NF membrane in NF-RO configuration. Due to the experimental constraints of this study, the specific energy requirements could not be presented in this paper.

## 4. Conclusions

Monovalent ions present in landfill leachates can permeate an NF membrane whereas organic solutes and other electrolytes are mostly rejected. Thus, both NF and RO stages in NF-RO can be operated at lower pressures compared to NF and RO stages in RO-NF. For similar operating conditions, individual stages in NF-RO provided higher water fluxes than those in RO-NF, showing NF-RO to be more energy efficient. Only a combination of biological and/or physical-chemical technologies together with membrane processes can fulfil complete and efficient treatment of landfill leachates. The ability of the NF stage in NF-RO to fractionate monovalent ions including NH_4_^+^ from other pollutants can be advantageous for realizing an effective and integrated system for treating a landfill leachate.

## Figures and Tables

**Table 1:** Solutes used for the preparation of 1 L synthetic leachate.

Solute	Weight (in g)	Solute	Weight (in g)
CaCl_2_·2H_2_O	0.84	NaHCO_3_	0.45
KCl	2.10	Na_2_SO_4_	0.83
MgCl_2_·6H_2_O	0.68	NH_4_HCO_3_	2.55
NaCl	6.73	Na-Humic acid	2.80

**Table 2:** Composition of raw leachate—major solutes (after analyses made by landfill operator).

**Ions (mg/L)**			
Ca^2+^	230	Mg^2+^	81
Cl^−^	5800	N-NH_4_^+^	580
HCO_3_^−^	3447	Na^+^	3100
K^+^	1100	SO_4_^2−^	560
**Sum parameters**			
pH	8.01	Conductivity (µS/cm)	23.5
COD (mg/L)	1900	TOC (mg/L)	840

COD—chemical oxygen demand, TOC—total organic carbon.

**Table 3:** Summary of experiments.

scheme 1 NF → RO	NF at 20 bar				RO at 60 bar			
	F = 200	R = 80	cf = 2.50	P = 120	F = 100	R = 50	cf = 2.00	P = 50
	NF at 30 bar				RO at 50 bar			
	F = 200	R = 77	cf =2.60	P = 123	F = 100	R = 47	cf = 2.13	P = 53
scheme 2 RO → NF	RO at 60 bar				NF at 20 bar			
	F = 220	R = 120	cf = 1.83	P = 100	F = 100	R = 74	cf = 1.35	P = 26
	RO at 50 bar				NF at 30 bar			
	F = 220	R = 130	cf =1.69	P = 90	F = 100	R = 28	cf = 3.57	P = 72

F—feed; R—retentate; P—permeate volume (in mL); cf—concentration factor.

**Table 4:** Measured parameters and analytical procedures.

Parameter	Instrument	Standard Method
Abs254	Jasco-V-550 UV–vis spectrophotometer	DIN 38404-3:2005-07
Ammonia-N	Jasco-V-550 UV–vis spectrophotometer	DIN 38406-5:1983-10
Conductivity	Greisinger-GLF 100 conductivity meter	DIN EN 27888:1993-11
TIC & TOC	Analytik Jena-Multi N/C 3000 analyzer	DIN EN 1484:1997-08
COD	Hach-LCK 314 cuvette/DR3900 photometer	DIN 38409-H41-H44

Abs254—absorbance at 254 nm; TIC—total inorganic carbon; TOC—total organic carbon; COD—chemical oxygen demand.

**Table 5:** Measured pure water fluxes, J_w_ (in L·m^−2^·h^−1^) and permeabilities, K_w_ (in L·m^−2^·h^−1^·bar^−1^) of the membranes at different operating pressures.

Membrane	OP = 25 Bar		35 Bar		55 Bar		65 Bar	
	J_w_	K_w_	J_w_	K_w_	J_w_	K_w_	J_w_	K_w_
NF270	306 ± 3	12.2 ± 0.1	373 ± 1	10.6 ± 0.1	-	-	-	-
SW30HR	-	-	-	-	80.4 ± 3.2	1.46 ± 0.06	77.4 ± 1	1.19 ± 0.01

**Table 6:** Measured parameters in feed, retentate and permeate samples from scheme 1—NF (20 bar) → RO (60 bar) and NF (30 bar) → RO (50 bar).

Parameter	Raw Leachate	NF Retentate		NF Permeate		RO Retentate		RO Permeate	
		P = 20 Bar	30	20	30	50	60	50	60
Abs254	0.15 * ± 0.01	0.32 *	0.37 *	0.05	0.04	0.10	0.07	0.01	0.01
TOC (mg/L)	398 ± 15	832	1046	<5	<5	<10	<10	<1	<1
TIC (mg/L)	286 ± 18	514	502	138	147	257	240	12	16
N-NH_4_^+^ (mg/L)	404 ± 28	488	600	294	315	595	530	27	18
Conductivity (mS/cm)	24.6 ± 0.1	29.8	32.8	18.2	17.5	34.6	33.9	0.36	0.34

* After 231 times dilution (otherwise without dilution).

**Table 7:** Measured parameters in feed, retentate and permeate samples from scheme 2—RO (50 bar) → NF (30 bar) and RO (60 bar) → NF (20 bar).

Parameter	Raw Leachate	RO Retentate		RO Permeate		NF Retentate		NF Permeate	
		P = 50 Bar	60	50	60	20	30	20	30
Abs254	0.15 * ± 0.01	0.24 *	0.27 *	0.01	0.01	0.27 *	0.72 *	0.44	0.09
TOC (mg/L)	398 ± 15	656	751	<1	<1	741	1972	n.a.	<10
TIC (mg/L)	286 ± 18	444	445	13	12	489	774	270	225
N-NH_4_^+^ (mg/L)	404 ± 28	688	668	29	16	649	932	555	543
Conductivity (mS/cm)	24.6 ± 0.1	37.6	42.5	0.41	0.36	40.3	49.2	36.0	30.2

* After 231 times dilution (otherwise without dilution); n.a.—not available.
